# Increased anxiety-like behavior is associated with the metabolic syndrome in non-stressed rats

**DOI:** 10.1371/journal.pone.0176554

**Published:** 2017-05-02

**Authors:** Daniela Rebolledo-Solleiro, Gabriel Roldán-Roldán, Daniel Díaz, Myrian Velasco, Carlos Larqué, Guadalupe Rico-Rosillo, Gloria Bertha Vega-Robledo, Elena Zambrano, Marcia Hiriart, Miguel Pérez de la Mora

**Affiliations:** 1 Division of Neuroscience, Instituto de Fisiología Celular, Universidad Nacional, Autónoma de México, Mexico City, Mexico; 2 Laboratorio de Neurobiología de la Conducta, Departamento de Fisiología, Facultad de Medicina, Universidad Nacional Autónoma de México, Mexico City, Mexico; 3 Departamento de Biología Celular y Fisiología, Instituto de Investigaciones Biomédicas, Universidad Nacional Autónoma de México, Mexico City, Mexico; 4 Laboratorio de Inmunología, Departamento de Medicina Experimental, Facultad de Medicina, Universidad Nacional Autónoma de México, Mexico City, Mexico; 5 Departamento de Biología de la Reproducción, Instituto Nacional de la Nutrición Salvador Zubirán, Mexico City, Mexico; Technion Israel Institute of Technology, ISRAEL

## Abstract

Metabolic syndrome (MS) is a cluster of signs that increases the risk to develop diabetes mellitus type 2 and cardiovascular disease. In the last years, a growing interest to study the relationship between MS and psychiatric disorders, such as depression and anxiety, has emerged obtaining conflicting results. Diet-induced MS rat models have only examined the effects of high-fat or mixed cafeteria diets to a limited extent. We explored whether an anxiety-like behavior was associated with MS in non-stressed rats chronically submitted to a high-sucrose diet (20% sucrose in drinking water) using three different anxiety paradigms: the shock-probe/burying test (SPBT), the elevated plus-maze (EPM) and the open-field test (OFT). Behaviorally, the high-sucrose diet group showed an increase in burying behavior in the SPBT. Also, these animals displayed both avoidance to explore the central part of the arena and a significant increase in freezing behavior in the OFT and lack of effects in the EPM. Also, high-sucrose diet group showed signs of an MS-like condition: significant increases in body weight and body mass index, abdominal obesity, hypertension, hyperglycemia, hyperinsulinemia, and dyslipidemia. Plasma leptin and resistin levels were also increased. No changes in plasma corticosterone levels were found. These results indicate that rats under a 24-weeks high-sucrose diet develop an MS associated with an anxiety-like behavior. Although the mechanisms underlying this behavioral outcome remain to be investigated, the role of leptin is emphasized.

## Introduction

Metabolic syndrome (MS) may be defined as a cluster of signs that increases the risk to develop diabetes mellitus type 2 (DM2), cardiovascular disease and some types of cancer. The cardinal signs of this condition are abdominal obesity, elevated blood pressure, dyslipidemia, and hyperinsulinemia with altered fasting glucose levels. Clinical diagnostic is determined when three or more of these signs are present [1].

In the last years there has been a growing interest to study the relationship between MS and psychiatric disorders, such as depression and anxiety. However, the results obtained so far have been rather contradictory. Carroll and colleagues [2] showed evidence for a positive association between general anxiety disorder and MS within a large population of male US veterans from the Vietnam War. Likewise, Weiss et al. (2011) [3] found that post-traumatic stress disorder [PTSD] is associated with increased rates of MS within an african-american urban population. More recently, Albert et al., [4] using a cross-sectional approximation, found that the prevalence of MS in a sample of patients with obsessive-compulsive disorder (OCD) is higher than those reported in the Italian general population. Additionally, in the last year, Tang and collaborators reported that anxiety has a significant positive association with MS using a meta-analytic approach [5]. In contrast with these findings, Hildrum et al., [6] did not find an association between anxiety and MS in a Norwegian cohort. Furthermore, in 2011, Linnville and collaborators [7] stated that the overall prevalence of MS was the same in repatriated prisoners of the Vietnam War with and without PTSD in comparison with a control group. Thus, it is clear that the information generated from human studies is not enough to clarify whether or not exists a relationship between MS and anxiety disorders [5] and the nature of this association.

For this reason, experimental strategies to model MS/DM2 based on changes in the amount of either fat or sugar (sucrose) in the diet of rodents (for a review see [8], [9]) have been successfully attempted. However, information on their usefulness as paradigms to study the relationship between these metabolic entities and anxiety is rather scarce and contradictory. Furthermore, such studies have been so far practically restricted to animals kept under a high-fat diet (HFD) and their results are depending upon the length of feeding and the presence of stress [10–13].

Accordingly, whereas mice kept under a medium-term HFD (either 6, 12 or 14 weeks) developed an anxiogenic-like profile in different anxiety paradigms [14–17], rats eating a similar diet for a week show instead an anxiolytic-like behavior in the elevated-plus-maze (EPM) [18, 19]. In addition, no effects in anxiety were however found when mice were evaluated after a moderate-term (13 weeks) HFD in a triple anxiety test (open-field test (OFT) + EPM + light-dark box test (LDBT) [20].

Studies on the effects of a high carbohydrate diet (HCD) on anxiety, on the other hand, have been less conclusive and differ from those obtained using the HFD model. Thus, in the same studies rodents under HCD usually composed of sucrose (producing glucose and fructose upon digestion) as a source of carbohydrate displayed anxiolytic effects in the OFT [20] but have no effects in the EPM [18]. In contrast, Santos and colleagues reported that mice under a HCD (composed of 10% of sucrose) for 12 weeks exhibited an anxiogenic profile in the contextual fear conditioning test and in the EPM (only when animals suffered 2 h of restraint stress) [21]. Also, rats who were fed with a high-fructose diet for 8 week showed anxiogenic effects in the OFT and the LDBT [22]. Interestingly, rats subjected to a powdered diet containing a moderate amount of sucrose (7.9%) failed to induce any change in anxiety-like behavior at 52 weeks, as compared with rats that were kept under a sugar-free diet [23].

Although in both models (HFD or HCD) there is an increase in the animal’s caloric intake, they may differ in the way MS is induced [9, 24] and perhaps in the mechanism linked to their behavioral consequences since fructose, which is produced by sucrose digestion has been reported to have a number of effects, independently of its caloric contribution [24, 25].

Recently, Larqué et al. [8] reported that the exposure of young adult Wistar rats to high-sucrose (20%) in drinking water for 8 weeks induces a MS-like condition, i.e., abdominal obesity, insulin resistance, elevated blood pressure and dyslipidemia. Moreover, when the animals are kept under the above experimental conditions for longer periods (24 weeks), a slight hyperglycemia is added to these signs [26].

It has been demonstrated that a chronic, low-grade inflammatory condition is developed during MS, which is characterized by an increased secretion of adipocyte-related cytokines (adipokines) such as leptin, adiponectin and resistin, and some other cytokines secreted by other tissues, including the brain [27–29]. Their plasma levels are inversely (adiponectin) or directly (resistin) correlated to obesity, and they have opposite effects on insulin sensitivity [27, 30]. Furthermore, obesity has been associated with a high prevalence of emotional reactivity and cognitive dysfunction [29, 31]. Some works have linked obesity and an increase in anxiety-like behavior in the EPM [32]. However, the possible role of leptin and resistin as a pathophysiologic mechanism in the generation of anxiety-like behavior is not completely understood.

Since MS is induced in rodents under a high-sucrose diet but changes on anxiety-like behavior are contradictory, the aim of this study was to ascertain whether a long-term high-sucrose diet (24 weeks) may be a useful rodent model to study the association between a MS-like condition and anxiety as well as its underlying factors. Metabolic and physiologic markers to ascertain whether or not MS was induced by the high-sucrose diet used were also measured.

## Materials and methods

All methods used in this study were approved by the Animal Care Comitee of the Instituto de Fisiología Celular, Universidad Nacional Autónoma de México (CICUAL-MPM06-14). Animal care was carried out according to the "International Guiding Principles for Biomedical Research Involving Animals", Council for International Organizations of Medical Sciences, 2010. Animal Research: Reporting of *In vivo* Experiments (ARRIVE) guidelines were also followed. Efforts were taken to minimize animals’ suffering throughout all experimental procedures.

One hundred and ninety six young adult male Wistar rats (250–280 g) bred in the Instituto de Fisiología Celular, Universidad Nacional Autónoma de México, Mexico City, were used. Rats were housed in a controlled environment (temperature 22°C, lights on 06:00–18:00 h), two animals per cage with food (Purina chow LabDiet 5001) and water/sucrose solution (20%) *ad libitum*. Somatometric parameters were evaluated in all animals. Further, randomly selected batches of animals were used to analyze behavior, cardiovascular, metabolic and hormonal traits. All rats were used only once.

### Metabolic syndrome model

**Fig 1:**
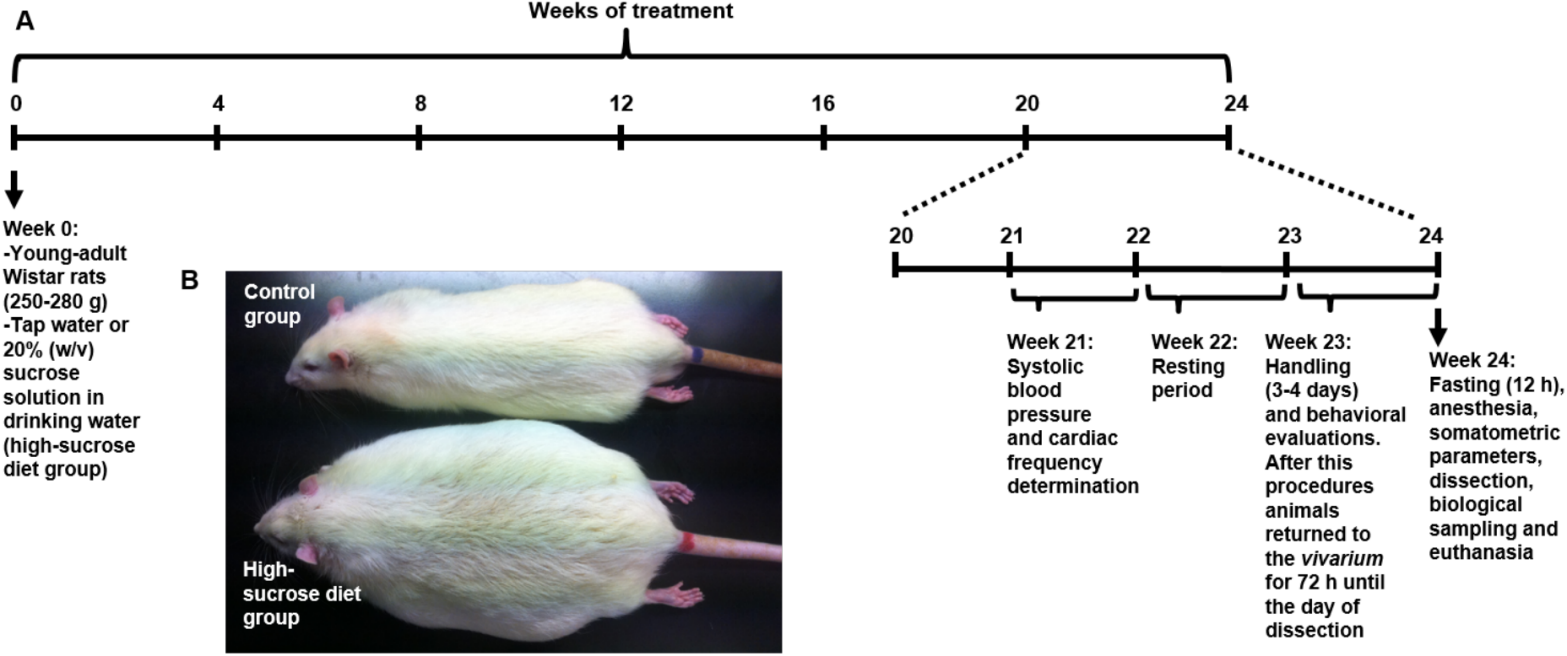
Experimental design. Timeline representing the experimental design utilized in this study; (B) representative picture of control and high-sucrose treated rats after 24 weeks of treatment.

In order to model MS, 8 weeks-old male rats (250–280 g) were submitted to a 20% (w/v) sucrose solution treatment that was provided as beverage *ad libitum* for 24 weeks, as reported by Velasco et al., [26]. Tap water was given to control age-matched rats. Both, high-sucrose diet and control rats were fed throughout the experiment with standard rat chow composed of 28.5% protein, 13.5% fat, and 58% carbohydrates. Behavioral evaluations were carried out during the last week of the high-sucrose diet. It is important to clarify that the high-sucrose diet group only had the choice to drink the 20% sucrose solution. Fig 1 describes the experimental design used in this study.

### Behavioral procedures

Behavioral evaluations were performed three days before obtaining biological samples to minimize stressful effects from the behavioral procedures over the metabolic parameters. It is worthy to mention that all animals that were evaluated behaviorally were also utilized to measure somatometric and metabolic parameters, such as glucose and insulin, whereas a sub-group of these animals was also evaluated for systolic blood pressure and cardiac frequency (see below).

Animals were handled 5 min once a day for 3 or 4 consecutive days before testing. Rats were kept overnight in the experimental room in order to avoid context influences and to keep their basal anxiety levels as low as possible. Behavioral experiments were conducted between 10:00 and 16:00 h in a sound-attenuated room equipped with video-recording facilities. The devices used for the evaluation of behavior were placed beneath the video camera, and behavior was recorded in the absence of any observer. In all the experiments rats were assigned to each group [control or high-sucrose diet] and tested in a randomized manner. To understand the effects of long-term high-sucrose diet on anxiety-like behavior, three different paradigms were chosen to evaluate both active (SPBT; n = 18 control group/n = 17 high-sucrose diet group) and passive coping responses (EPM and OFT; n = 27 control group/n = 24 high-sucrose diet group) in our animals. Taking into account that a previous behavioral evaluation may influence the outcome of a subsequent test [33–35], and even have unpredictable effects on the state of some important receptors [36], EPM was carried out in animals that have never been behaviorally evaluated.

#### Shock-probe/burying test

The test was carried out as described by Treit et al. [37] and Fernández-Guasti et al. [38]. The test was conducted in an acrylic cage (27 x 16 x 23 cm) with its floor covered with a uniform layer (5 cm) of fine sawdust. The cage was equipped with an electrified probe (7 cm long, 0.5 cm thick), which protruded from one of its walls 5 cm above the bedding and through which the rats received an electric shock (0.3 mA) any time they come into contact with the probe. The current was generated by a constant current shocker (LaFayette Instruments, Inc). During the test four parameters were recorded: the total amount of time that the rat spends burying the probe either with the forepaws or with the hind paws (burying behavior), the total amount of time that the rat spends lying or sitting with the body motionless except for tiny and slow lateral movements of the head or the ones needed for breathing (freezing behavior), time elapsed from the first shock to the start of the burying behavior (burying behavior latency), and the number of shocks received by the rat during the test.

#### Elevated plus-maze

The maze used in this work was based on the design of Pellow et al. [39] and was constructed as previously described [40] with slight modifications to allow obese rats to move freely. The maze consisted of two open arms (50 x 13 cm) divided in three quadrants and two enclosed arms (50 x 13 x 40 cm) with an open roof. The arms intersected at the central square (13 x 13 cm). The maze was elevated 50 cm from the floor by a pedestal joined to the central square. In order to avoid rats falling from the maze, wooden ledges (0.5 x 0.5 cm) were attached along the edges of the open arms. With the purpose to study to what extent animals explore the open arms of the maze, lines dividing its full length in three equal quadrants, were painted on their floor (see diagram in Table 1). Rats were placed on the central square of the maze facing an open arm at the beginning of the test, and were allowed to explore the maze for 5 min. The number of entries to the open arms (expressed as the percentage of the total number of arm entries), and the total time spent in these arms of the maze were taken as an anxiety index (the higher the index the lower anxiety). The total number of arms (open+closed) entries was taken as a measure of locomotion [39, 40]. An entry was counted when the four paws of the rat were placed in the respective arm. The maze was cleaned with detergent and dried before each trial. The illumination level at the central square of the maze was 5.1 lux during testing.

**Table 1:** Somatometric parameters measured in control and high-sucrose diet groups.

	Control group	High-sucrose diet group
Body weight (g)	551 ± 13	662 ± 13 ***
Body length (cm)	27 ± 0.2	27 ± 0.2
Body mass index (Kg/m)^2^	7.7 ± 0.2	9 ± 0.2 ***
Abdominal circumference (cm)	22 ± 0.3	25 ± 0.3 ***
Total abdominal fat (g)	8.2 ± 0.5	18 ± 0.8 ***

Mean ± SEM; Student T-test

*** P<0.001

#### Open-field test

Since time spent in the center of the open field is considered a measure related to anxiety [41], in order to further analyze this behavior, high-sucrose diet group and control rats were placed in an open field apparatus 1 min after being evaluated in the EPM. Evaluations were carried out in an arena (50 x 50 x 30 cm) divided into 25 squares (10 x 10 cm) as previously described [40]. Each rat was placed in the center of the arena and was allowed to explore it for 5 min. The time spent in the central squares of the arena, as well as the time that the rat spent in freezing behavior was quantified “off-line”. Freezing behavior was defined by the total amount of time that the rat spends lying or sitting with the body motionless except for tiny and slow lateral movements of the head or the ones needed for breathing. For measuring locomotion photoelectric cells were used to record the horizontal movements of the animals through the arena (OMNIALVA, Mexico City, Mexico). Each wall contained 10 photoelectric cells separated by 5 cm from each other that were located 4.0 cm above the arena. The box was interphased with a PC that allowed estimating quantitatively the distance traveled by the animal. Light intensity at the center of the arena was 138 lux.

### Somatometric parameters

Somatometric and metabolic measurements were used to assess MS-like condition as reported previously [26]. Twenty-four weeks after high-sucrose diet, animals were fasted for 12 h and anesthetized with an intraperitoneal sodium pentobarbital (40 mg/kg) injection to obtain biological samples. The following parameters were measured, body weight, length (from the tip of the nose to the anus) and abdominal circumference (the diameter of the abdominal region just above the iliac crest). Body mass index (BMI) was calculated as body weight (Kg)/length (m) ^2^. While the rats were still anesthetized, blood was taken from the inferior cava vein. Abdominal fat samples (peripancreatic and epididymal adipose tissue) were also obtained and weighted. After sample collecting, animals were euthanized by cervical dislocation.

### Blood pressure

Blood pressure was measured in a sub-group of rats evaluated behaviorally (control group: n = 26/ high-sucrose diet: n = 26) between 21 and 22 weeks of treatment. The tail-cuff procedure was used to obtain systolic blood pressure (SBP) with a tail-cuff equipment (Landing, NJ USA). Animals were trained in several sessions before to practice the SBP measurement, which was done in awake rats. We performed an average of five readings to obtain individual SBP values. Normal SBP was considered between 110–135 mm Hg, while hypertension was diagnosed when values were above 140 mm Hg.

### Metabolic parameters

Blood samples from fasted animals were collected from the inferior cava vein in heparinized tubes and immediately centrifuged (1500 rpm during 10 minutes at 4°C) to obtain plasma. Samples were stored at -20°C until its posterior analysis. Plasma insulin level (n = 45 control group/n = 45 high-sucrose diet group) was determined with a Mercodia ultrasensitive rat insulin ELISA system and plasma glucose (n = 45 control group/n = 50 high-sucrose diet group) was measured with a glucose analysis kit (Quantichrom, BioAssay Systems, Hayward, CA USA) as described before [26]. Separate groups of rats, not evaluated behaviorally but otherwise treated and handled in the same way were used for measuring: leptin, resistin, triglycerides and corticosterone. Triglycerides (n = 26 control group/n = 38 high-sucrose diet group) were determined by a photometric method (assays kits were manufactured by Beckman Coulter, Inc.). Levels of serum resistin and leptin (n = 26 control group/n = 38 high-sucrose diet group) were measured by enzyme-linked immuno-sorbent assay (ELISA) with commercial kits: rat resistin ELISA kit (GenWay, Biotech, Inc. USA) and mouse leptin ELISA kit (R&D Systems, Inc. Minneapolis, USA), according to the manufacturers´ instructions, as previously reported [42–44]. Basal plasma corticosterone levels (n = 20 control group/n = 18 high-sucrose diet group) were quantified by radioimmunoassay [45] as using the Coat-A-Count® rat corticosterone kit and following the indications of the manufacturer.

### Statistical analysis

All data are expressed as means ± SEM. Normal distribution was tested using the Kolmogorov-Smirnov test. Changes in either behavioral outcomes or somatometric and metabolic parameters were evaluated using the Mann-Whitney test and the Student´s t-test, respectively. Fisher´s exact test was used to compare the extent of open arm exploration between groups. Significance was set at p<0.05. Statistical parameters were computed using GraphPad Prism 5 statistical software.

## Results

### Behavioral evaluation

#### Shock-probe/burying test

**Fig 2:**
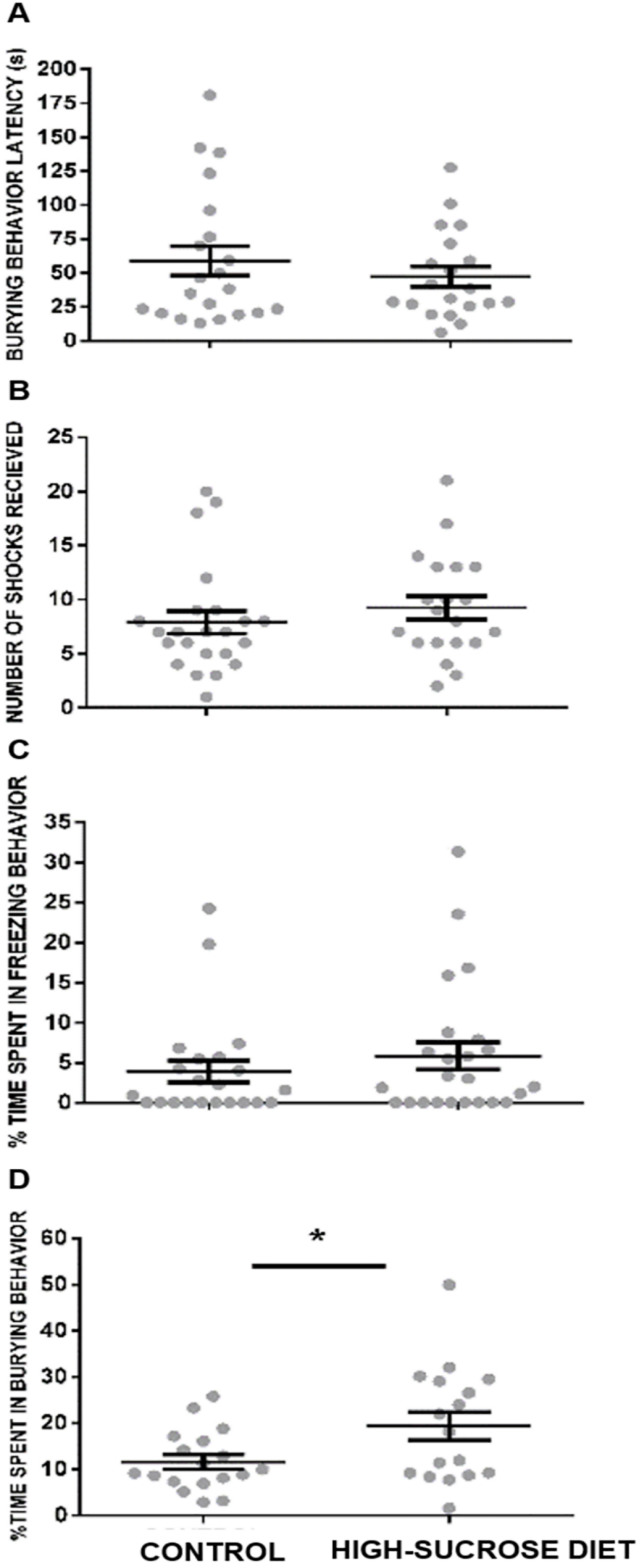
Effect of a 24 week high-sucrose diet in rats evaluated in the shock-probe/burying test. A significant increase was noticed in the group submitted to a high-sucrose diet in time (%) spent in burying behavior (C) when compared with the control group. No significant effects were elicited on burying behavior latency (A), number of shocks received (B) and the percentage of time spent in freezing behavior (D). Bars represent means ± SEM. Mann-Whitney Test was conducted, *p<0.05. Control group n = 18, high-sucrose diet group n = 17.

As indicated in Fig 2, the sucrose-treated group spent significantly more time (%) displaying burying behavior when compared to the controls (U = 93, p<0.05) (Fig 2C). No effects were elicited in the other parameters measured during testing. Thus, neither latency to bury (U = 202, p = 0.8449) (Fig 2A), total number of shocks received (U = 181.5, p = 0.2405) (Fig 2B) nor the percentage of time spent in freezing behavior (U = 226, p = 0.3924) (Fig 2D) were affected. It is important to mention that the animals that did not display burying behavior were excluded of this analysis (control group = 5/23, high-sucrose diet group = 3/20).

#### Elevated plus-maze

**Fig 3:**
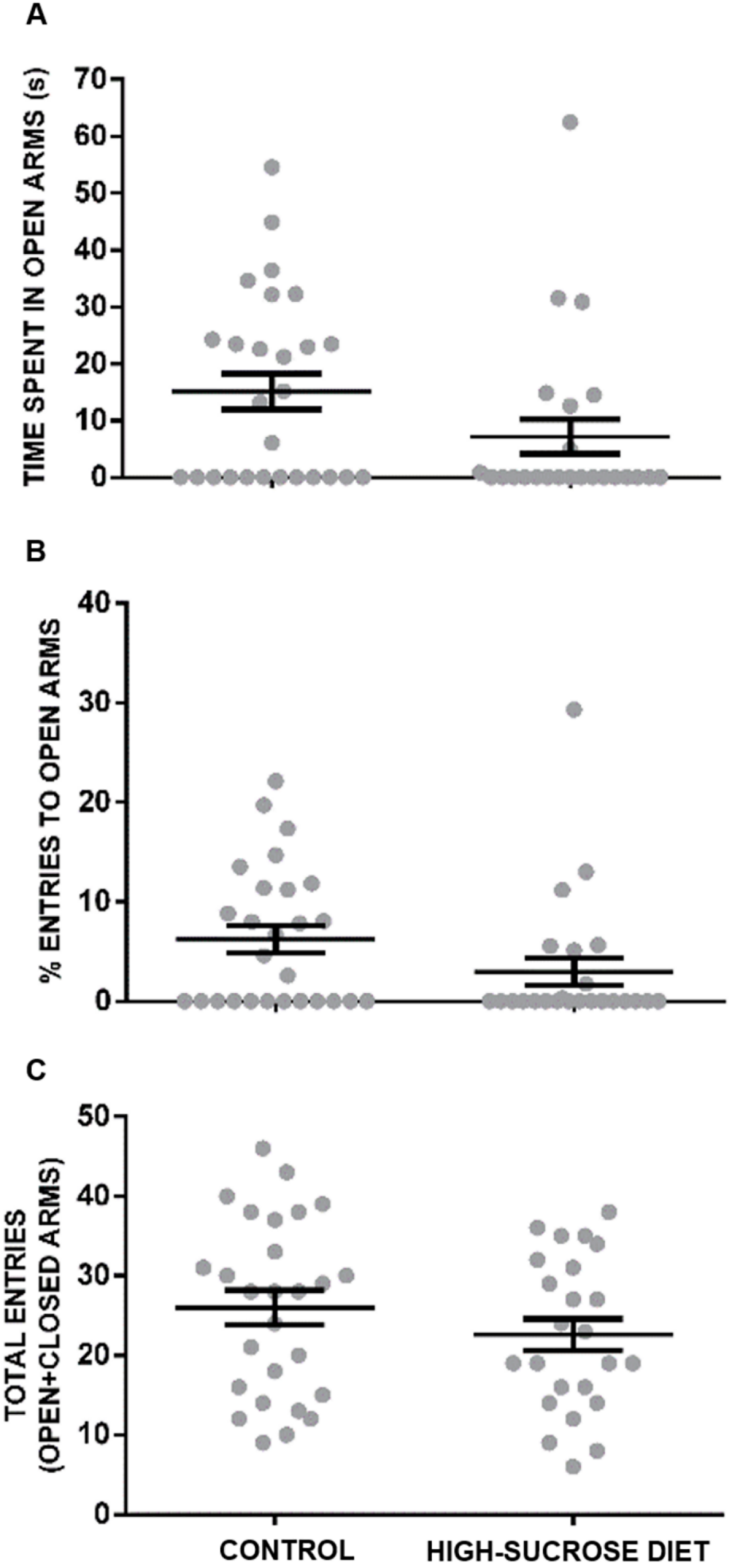
Effect of a 24 week high-sucrose diet on anxiety-like behavior in the elevated plus-maze. Although not significant, the high-sucrose diet group showed a nonsignificant trend (U = 230, P = 0.053) for a reduced time spent in open arms (A) and a lower percentage of open arms entries (B) when compared with the controls. No effects were observed in the total number of entries into the open+closed arms of the maze (C). Bars represent means ± SEM. Mann-Whitney Test was conducted. Control group n = 27, high-sucrose diet group n = 24.

Sucrose-treated rats displayed a non significant trend (U = 230, P = 0.053) for decrease time spent in the open arms (Fig 3A) as well as fewer open arm entries when compared (U = 230; P = 0.0534) to the control group (Fig 3B). No effects were observed in the total number of entries into the open+closed arms of the maze (U = 270, p = 0.2861) (Fig 3C).

**Fig 4:**
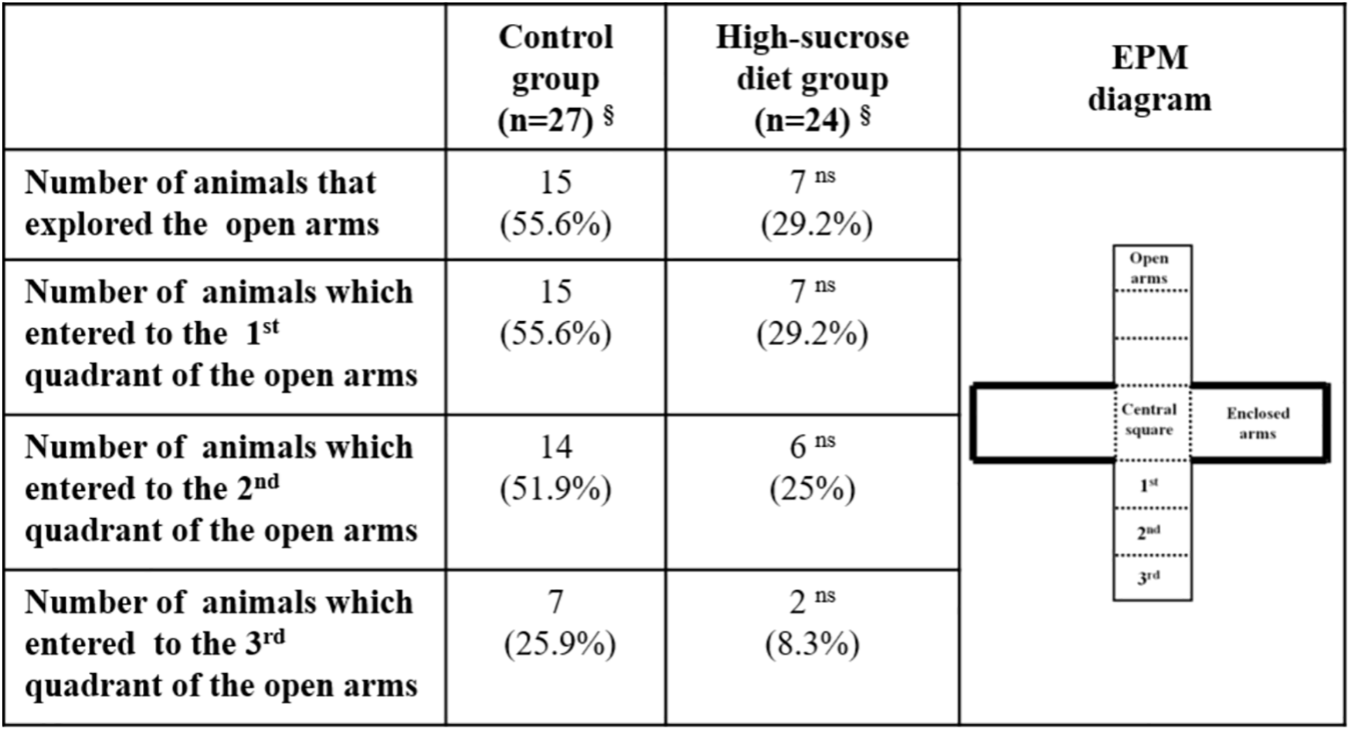
Qualitative analysis of the behavior displayed by control and high-sucrose diet groups in the elevated-plus maze.

A further analysis of the behavior displayed by rats in the EPM (Fig 4) showed that a higher percentage of animals within the high-sucrose diet group did not explore at all the open arms of the maze when compared with the control group (control group: 44.4% vs. high-sucrose diet group: 70.8%). Moreover, the number of animals, that explored the second and third quadrants of the open arms of the maze, was lesser within the high-sucrose diet group in comparison with the control group (second quadrant: 25% vs. 51.9%; third quadrant: 8.3% vs. 25.9%, respectively; p>0.05 Fisher´s exact test).

#### Open-field test

**Fig 5:**
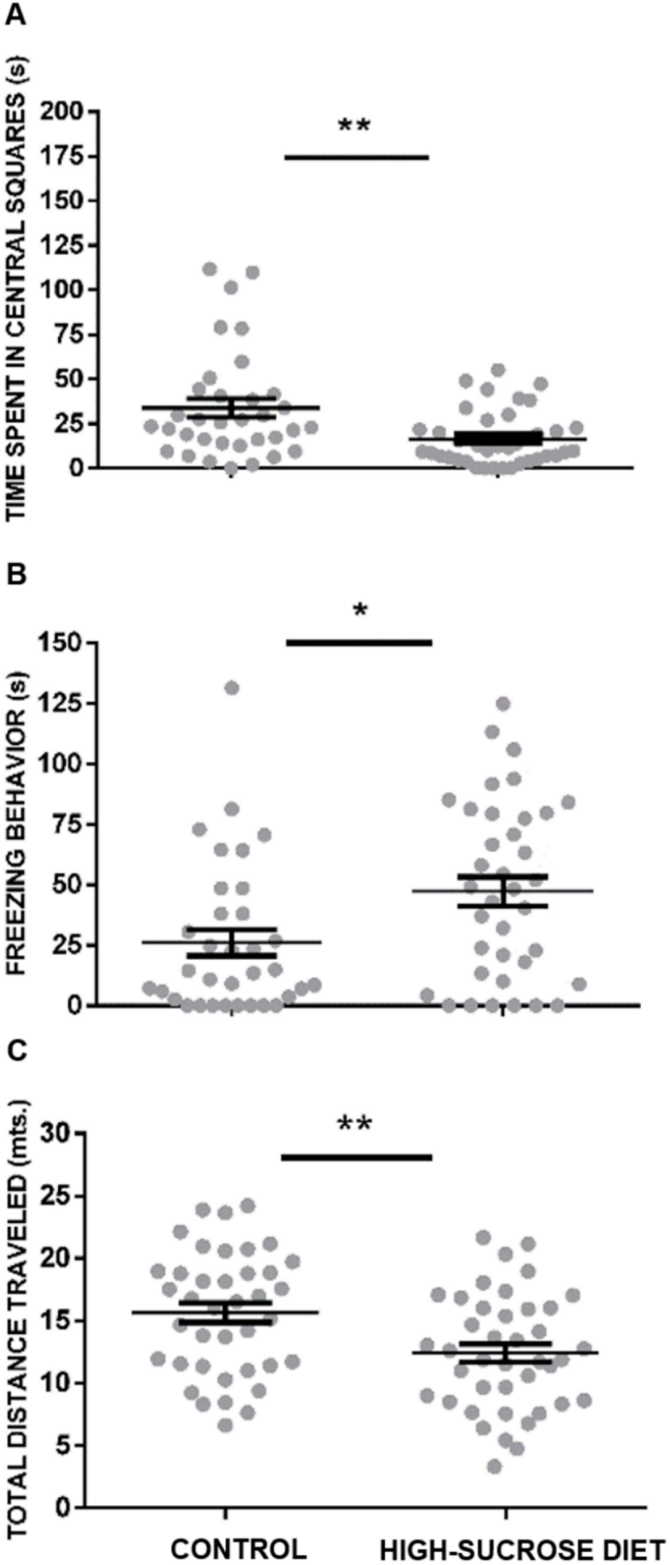
Effect of a 24 week high-sucrose diet on anxiety-like behavior as measured in the open-field test. The high-sucrose diet group exhibited a reduction in the time spent in the central squares (A) and an enhancement in freezing behavior (B) as compared with the control group. Moreover, a decrease of the total distance traveled by the rats within the open-field was observed in the group submitted to the high-sucrose diet (C). Bars represent means ± SEM. Mann-Whitney Test was conducted, **p<0.001. Control group n = 27, high-sucrose diet group n = 24.

As shown in Fig 5A, the high-sucrose diet group exhibited a significant reduction in the time spent in the central squares of the open field, as compared with the control group (U = 194, p<0.001). Moreover, a significant increase in freezing behavior was observed in rats subjected to the high-sucrose diet (U = 149, p<0.05) (Fig 5B). In line with this, a decrease in the total distance traveled within the open-field was registered in the group within the high-sucrose diet in comparison with the controls (U = 260, p<0.001) (Fig 5C).

### Somatometric parameters

Table 1 shows somatometric changes elicited by a high-sucrose diet for 24 weeks. This experimental manipulation resulted in a significant increase in body weight (t = 5.9, df = 136; p<0.0001) and body mass index (t = 5.1, df = 136; p = <0.0001) when compared with the age-matched control group. Moreover, animals submitted to this treatment showed an increase in total abdominal fat (peripancreatic+epidydimal) (t = 9.0, df = 136; p<0.0001), which also caused a significant rise in the abdominal circumference (t = 5.6, df = 136; p<0.0001) as compared to the controls.

### Metabolic parameters

**Fig 6:**
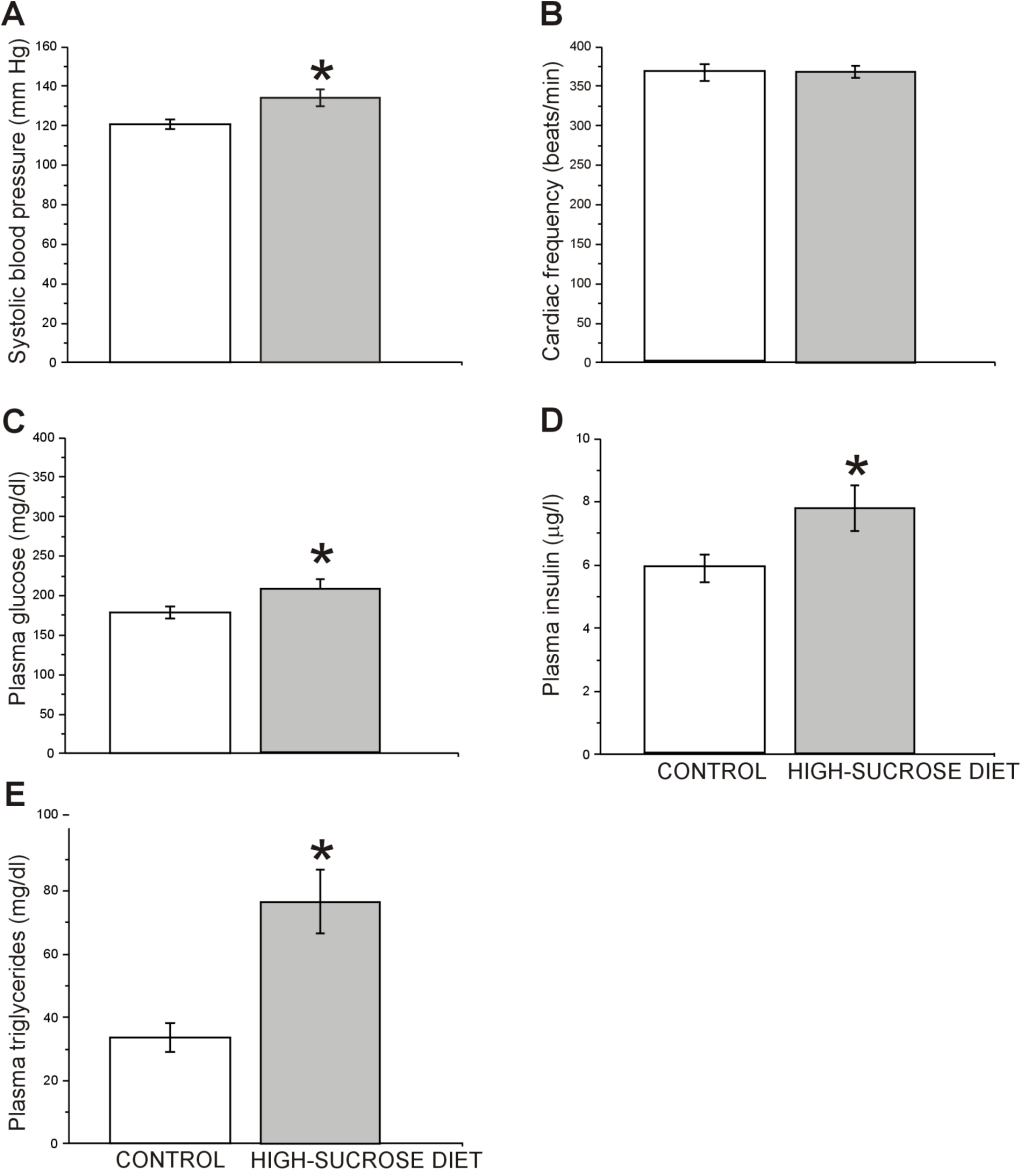
Effect of a 24 week high-sucrose diet on metabolic parameters. High-sucrose diet rats showed a significant increase in systolic blood pressure (A), without changes in cardiac frequency compared to control rats (B) (n = 26 for high-sucrose and control groups). Moreover, a significant increase in plasma insulin (D) (n = 45 for high-sucrose and control groups), glucose (C) (control group n = 45/high-sucrose diet group n = 50) and triglycerides (E) (control group n = 26/high-sucrose diet group n = 38) levels was observed in the high-sucrose diet group with respect to the controls. Bars represent means ± SEM. Unpaired, two-tailed Student t-test was conducted, *p<0.05, **p<0.01. Control group n = 43, high-sucrose diet group n = 46.

As depicted in Fig 6, we observed that systolic blood pressure (Fig 6A) was significantly increased in the high-sucrose diet group (t = 2.8, df = 50; p = 0.0066) without changes in cardiac frequency (t = 0.036, df = 50; p = 0.9716, Fig 6B) when compared to the control group. In addition, a significant increase in plasma levels of insulin (t = 2.2, df = 87; p = 0.0341, Fig 6D), glucose (t = 2.0, df = 93; p = 0.0450, Fig 6C) and triglycerides (t = 3.8, df = 87; p = 0.0341, Fig 6E) was observed in the high-sucrose diet group compared to the controls.

**Fig 7:**
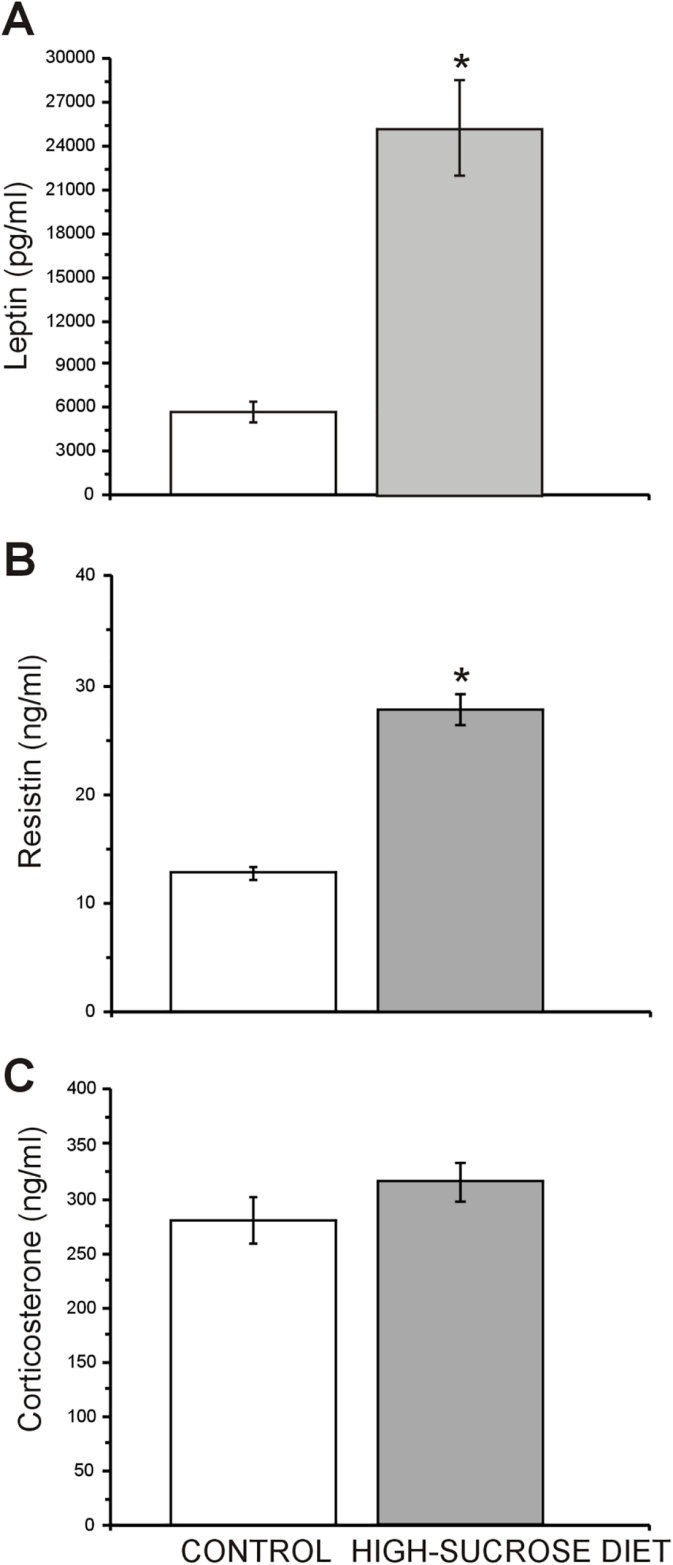
Effect of a 24 week high-sucrose diet on hormonal traits involved in metabolism and stress. Plasma leptin (A) and resistin (B) levels in the high-sucrose diet group were significantly elevated compared with the controls (n = 26 control group/high-sucrose diet group n = 38). Plasma corticosterone levels (C) did not show statistical difference between groups (n = 20 control group/high-sucrose diet group n = 18). Bars represent means ± SEM. Unpaired, two-tailed Student t-test was conducted. *p≤0.05.

To further investigate the role of some hormones involved in metabolism that could be related with anxiety-like behaviors, another batch of animals not evaluated behaviorally was used to evaluate plasma levels of corticosterone, leptin and resistin. As seen in Fig 7 both, plasma leptin (t = 5.15, df = 58; p<0.0001; Fig 7A) and resistin (t = 8.8, df = 58; p<0.0001; Fig 7B) levels were elevated in the high-sucrose diet group as compared to the control group. In addition, in spite that both anesthesia and the sampling procedure used in this work may have increased our basal plasma corticosterone levels [46], no statistical difference between control and experimental groups was found in post-anesthetized plasma corticosterone levels (t = 1.5, df = 35; p = 0.1457; Fig 6C).

## Discussion

The main finding of the present study is the observation, for the first time, of an anxiogenic-like profile in non-stressed adult rats kept under a high-sucrose diet, which is added to the alterations observed in animals with MS.

As reported by Velasco et al. [26] rats drinking a high-sucrose solution (20%; w/v) *ad libitum* for 24 weeks develop a MS-like condition, characterized by body weight gain, abdominal obesity, hyperglycemia, insulin resistance, dyslipidemia and elevated blood pressure, which altogether resembles what in humans is considered MS [1]. Moreover, hyperleptinemia and increased plasma resistin levels were also observed.

The anxiogenic-like profile observed in rats subjected to a high-sucrose diet in this study is evidenced by an increase in burying behavior in the SPBT, which is considered a sign of anxiety [40, 47]. In addition, in the OFT an avoidance to explore its central part and an increase in freezing behavior was noticed. Finally, a non-significant trend to reduce both, the number of entries and the time spent into the open arms of the EPM, was also observed. In line with the latter trend, rats kept under the high-sucrose diet explored less frequently and less thoroughly the open arms of the EPM than the controls. The lack of undisputed statistically significant anxiogenic-like changes observed in the EPM in this study may have arisen as a consequence of a “ceiling effect”, since under our experimental conditions a considerable percentage of animals within the control group (44%) did not explore at all the open arms of the maze making uncertain the comparison between control and high-sucrose diet groups.

Our behavioral results are in accordance with the observations made by Reddy and colleagues who showed that Sprague-Dawley rats that consumed a HCD (65% fructose in drinking water) for 8 weeks displayed anxiogenic-like behaviors in the OFT and the LDBT [22]. They also agree with the findings obtained by Santos et al., who reported an anxiogenic profile in mice under a HCD (10% of sucrose for 12 weeks) in the contextual fear conditioning test, and a lack of effects in the EPM when animals were non-stressed [21]. Nevertheless, our results differ from other studies which reported no effects in the EPM when rats are subjected to a high-sucrose diet for 7 days [18]. They also contrast with the observations made in mice submitted to a high-sucrose diet for 13 weeks, which showed anxiolytic effects in the OFT [20]. Moreover, no effects in the EPM were observed in rats that consumed a long-term moderate sucrose-diet (7.9%) [23], or in the marble burying test when mice were fed with a HCD (35% sucrose) for 4 weeks after a period of seven days where animals were replaced with a standard diet [48]. These differences are difficult to reconcile with our findings and among themselves, but they may be linked to differences in species (mice vs. rats), rat strain (Wistar vs. Sprague-Dawley) and/or to different experimental procedures (i.e. anxiety models, previous stress and/or time of high-sucrose diet exposure). In this regard, it is interesting to highlight that whereas in the work of Prasad and Prasad [18] rats were exposed to a high-sucrose diet for 8 days and in the other laboratories mice were fed with carbohydrate-rich diet for either 4 [48], 12 [21] or 13 weeks [20], in our work rats were treated with a high-sucrose diet for 24 weeks. Thus, it may be possible that anxiogenic effects will be only developed when high concentrations of sugars are used in their diets and/or after a long-lasting high-sucrose diet exposure. In agreement with this, we did not find any effects on anxiety-like behavior when rats were treated with a high-sucrose (20%) diet for only 8 weeks (data not shown).

The mechanisms involved in the increase of anxiety-like behavior in the high-sucrose diet group observed in this work is unknown. However, since protein glycation has been suggested to underlay the late effects of diabetes through the formation of advanced glycation end-products (AGEs) [49], it may have had a role in the development of the anxiogenic-like profile observed in our experiments. In line with this, we were unable to detect anxiogenic effects at 8 weeks after the high-sucrose diet (data not shown), but they were evident at 24 weeks, when the hyperglycemic condition was already established [26]. In support of this idea, Gancheva and Zhelyazkova-Savova [50], reported that rats modeling MS (animals subjected to a high-fat+high-fructose diet for 10 weeks), exhibited an anxiogenic-like profile in the social interaction test, which was reverted when administering vitamin K2 by gavage, which also normalized blood glucose levels. In view of this, it will be considerably interesting for the future to study whether or not both glycated hemoglobin and AGESs levels will increase following a long-term high-sucrose diet, and to see whether they bear any relationship to anxiety.

The possibility that the increase in anxiety-like behavior observed in the high-sucrose diet group may be a consequence of the hypertension shown by these animals also seems unlikely, because elevated blood pressure is already present 8 weeks after the beginning of the sucrose treatment [8], long before they develop anxiety-like behavior.

Although obesity/MS/mood disorders bear a complicated relationship is unlikely that dysregulated anxiety increases the risk for MS, since to our knowledge there is no epidemiological evidence that shows that anxious patients show a major risk to develop MS/DM2 in comparison to the general population. In fact, data from our group indicates that anxiety-like behavior in the EPM, the OFT and the SPBT, is not apparent at 8 weeks of treatment (data not shown), at a time when most signs of the MS were already present [8], indicating that those signs were not drove by anxiety.

It is worthy to note that although consumption of high-energy diets has been considered a background form of chronic stress [51] and hypothalamus-pituitary-adrenal (HPA) activation has been associated with an increase of anxiety [52–54], no effects in corticosterone post-anesthesia levels were found in this work. These findings suggest that under our experimental conditions anxiety did not emerged as a consequence of HPA activation. It is important to mention that to the best of our knowledge, none of the studies that used HCD (high-sucrose or high-fructose) have evaluated plasmatic corticosterone levels, with the exception of our study. Thus, there are not data available to compare our results with previous reports. Interestingly, the lack of effects observed on post-anesthesia corticosterone levels in this work are also in agreement with the results of Sharma and Fulton [15] and Yamada et al. [55] who also failed to find any change in basal corticosterone levels in non-stressed rats after a HFD. This observations differ from the results of Tannenbaum et al. [51] who reported elevated basal corticosterone levels as early as 7 days from the onset of a fat-rich diet and remained elevated for at least 21 days. They also contrast with the results obtained by Kurhe, et al., who showed increased plasma corticosterone levels in non-stressed mice under a HFD for 14 weeks [16]. The reasons for these differences are not clear, but they may be related to the length of exposure of the animals to the high caloric diet, to diet composition and to the action of compensatory mechanisms [10, 56]. Thus, whereas in our experiments rats were exposed 24 weeks to the high-sucrose diet, in the case of Sharma and Fulton [14], Kurhe [16, 17] and Yamada et al. [55] for 12, 14 and 16 weeks respectively, and in the work of Tannenbaum et al. [51] rats were only exposed for 21 days.

On the other hand, plasma leptin, an adipocyte-derived hormone encoded by the *ob* gene, was found elevated in this work. Since leptin deficient mice (ob/ob) exhibit an anxious phenotype [57], and acute leptin administration produces both anxiolytic effects in normal mice [58] and attenuates anxiety in ob/ob mice [59], it may be feasible that leptin resistance may have developed as a consequence of the high-sucrose diet and be involved in the anxiogenic effects reported in this paper. In support to this possibility, it has been reported that diet-induced obese rats show a self-resistance state to leptin within the hypothalamus [60, 61] and the ventral tegmental area (VTA) [62, 63]. This condition may have been evolved through a diminished leptin receptor gene expression accompanied by reduced signal transduction [61] and/or a putative reduction in leptin transport into the cerebral-spinal fluid [64]. On the other hand, it is generally admitted that amygdala plays a paramount role in the modulation of anxiety [65] and that considerable experimental evidence indicates that dopaminergic receptor mechanisms play an important role in this modulation [66, 67]. Since VTA neurons are the origin of the dopamine (DA) innervation of the amygdala [67] and it has been demonstrated that conditional knock-out mice lacking leptin receptor (Lepr^dat-Cre^ mice) in the VTA show an anxiogenic profile [58], it is conceivable that leptin resistance within VTA may underlie the anxiety behavior observed in our experiments. Moreover, it is tempting to speculate, that such anxiogenic effects may be mediated through activity of dopamine D1 receptors since the intra-amygdaloid administration of SCH-23390, a selective D1 receptor antagonist, attenuated anxiety in Lepr^dat-Cre^ mice [58] and induces anxiolytic responses in rats subjected both to the LDBT [67], the EPM [68] and the SPBT [69].

Resistin secreted by adipocytes is related to insulin resistance in rodents. Nevertheless, its association with cardiometabolic disease in humans is less defined [70]. In the last few years, it has been reported that plasma resistin levels are raised in patients with OCD and that this finding may account for cardiovascular and metabolic abnormalities seen in them [71]. Moreover, there are some evidences showing that resistin, similarly to leptin, can affect the central mechanisms of feeding by inhibiting catecholamine release in the hypothalamus [30], suggesting that it could also modulate other behaviors. In agreement with our results obtained in the high-sucrose diet group, obese subjects have shown higher resistin concentrations than non-obese controls [72, 73]. Also in accordance with our findings, higher levels of resistin were found in the db/db mice [74], which also models MS [8]. Since the role of this adipokine on anxiety and depression has been scarcely explored [75], further investigation is needed to determine the possible role of leptin resistance or hyperleptinemia and increased plasma levels of resistin in the increased anxiety-like behavior observed in our MS model.

It is interesting that recent studies have suggested that the composition of the intestinal microbiota have a strong impact in the modulation of several physiological processes [76] that include affective responses [20, 77], and that its composition is modulated by diet [78] and obesity [79]. Remarkably, gut microbiota composition is altered in mice submitted to a HFD for 13 weeks [20] and in the db/db mice [80]. However, the possibility that changes in the gut microbiota composition following treatment with 20% sucrose had been responsible for the behavioral changes reported here seems unlikely, since no differences in the composition of the intestinal microbiota were reported in mice exposed to a long-lasting high-sucrose diet [20]. In support of this idea, a chronic prebiotic treatment (8 weeks) in db/db mice improved metabolic parameters in these animals, but failed to reverse the anxiety-like profile observed on them [74]. Nevertheless, future studies to assess this relationship in our model are still needed, since it has been established that the microbiome influences anxiety and depression [81, 82].

Finally, since depression-like behavior has been reported in rodents kept on a long-lasting HFD [14, 15, 20, 55] and there is a high co-morbidity between anxiety and depression, but this work is mainly concerned with anxiety, it remains for the future to establish whether rodents on a long-lasting sucrose-rich diet will also develop behavioral signs of depression.

In conclusion, the results of this study support the notion that chronic high-sucrose diet induces a MS-like condition in rats, and that there exists a close relationship between this pathological entity and anxiety-like behavior. Moreover, our results suggest that a chronic high-sucrose diet may be useful to study the factors involved in this link. It is however clear that further studies will be needed to disclose the nature of the actual mechanisms underlying such an association.
